# A pan-cancer analysis of the oncogenic role of dual-specificity tyrosine (Y)-phosphorylation- regulated kinase 2 (DYRK2) in human tumors

**DOI:** 10.1038/s41598-022-19087-7

**Published:** 2022-09-14

**Authors:** Xinyue Qiu, Cheng Shen, Wenjing Zhao, Xunlei Zhang, Dakun Zhao, Xuming Wu, Lei Yang

**Affiliations:** 1 Cancer Research Center Nantong, Affiliated Tumor Hospital of Nantong University, 30 Tongyang North Road, Nantong, 226361 China; 2 Department of Oncology, Affiliated Tumor Hospital of Nantong University, 30 Tongyang North Road, Nantong, 226361 China; 3 Affiliated Mental Health Center of Nantong University, 37 Chenggang Road, Gangzha District, Nantong, 226005 China; 4 Department of Computer Science and Engineering, Tandon School of Engineering, New York University, Brooklyn, NY 11201 US

**Keywords:** Cancer, Oncology

## Abstract

Although there have been studies correlating DYRK2 with a number of human cancers, there has been no pan-cancer analysis. Therefore, through the TCGA database, we conducted a related study on the expression of DYRK2 in cancers.The expression of DYRK2 is obviously increased in some cancers, while the opposite is true in others, and there is a clear association between its expression and the prognosis of cancer patients.The mutation of DYRK2 is also significantly correlated with patients’ prognosis in certain human tumors. In addition, phosphorylation and methylation levels of DYRK2 are different between tumor tissues and adjacent normal tissues in various tumors. In the tumour microenvironment, the expression of DYRK2 correlates with cancer-associated fibroblast infiltration, such as BLCA or HNSC. In order to fully understand the role of DYRK2 in different tumors, we conducted a pan-cancer analysis.

## Introduction

Since tumor development is a complex and variable process, exploring the underlying molecular mechanisms of related genes through a pan-cancer analysis is of great significance for improving patient prognosis and enhancing clinical outcomes.The public funded project TCGA(The Cancer Genome Atlas) and the GEO (Gene Expression Omnibus) have involved functional genomics datasets of a variety of tumours to help complete the pan-cancer analysis^1–3^.

Dual-specificity tyrosine (Y)-phosphorylation- regulated kinase 2 (DYRK2) belongs to the DYRK family,which is a member of the CMGC (cyclin-dependent kinases, mitogen-activated protein kinases, glycogen synthase kinases and CDC-like kinases) superfamily^4^. This kinase autophosphorylates tyrosine residues but acts as a serine/threonine kinase on its substrate^5^. DYRKs are traditionally divided into two groups: the first group (DYRK1A and DYRK1B) and the second group (DYRK2, DYRK3, and DYRK4), which all contain serine and threonine sites for phosphorylating substrates.There is evidence that DYRKs regulate cell function and are involved in cell survival, cell differentiation, gene transcription, and endocytosis. The first group of DYRKs are mainly located in the nucleus, while the second group of DYRKs are mainly located in the cytoplasm. Therefore, DYRK2 is mainly expressed in the cytoplasm^6,7^. It has oncogenic and antitumor activities in several human cancers^8–10^. And most of all, in the case of DNA damage, DYRK2 in the cytoplasm is phosphorylated by the activated ATM (ataxia- telangiectasia mutant group), which translocates into the nucleus and phosphorylates p53 at Ser46^11^. On the other hand, ATM phosphorylates DYRK2 in the nucleus at Thr-33 and Ser-369, thus allowing nuclear DYRK2 to avoid degradation by ubiquitination through dissociation from the E3-ubiquitin ligase double minute 2 (MDM2). The phosphorylation of p53 at Ser46 leads to apoptosis^12^.

DYRK2 plays different roles in cell growth, proliferation and development, with a focus on its role in cancer^13–17^. For example, recent studies have found that DYRK2 is a major regulator of the hitherto unknown heat shock factor 1(HSF1) pathway, promoting triple-negative breast cancer(TNBC) cell survival^18^.However there is still a need to demonstrate the relationship between DYRK2 and various tumours through clinical big data analysis.

This research performed extensive cancer analyses of DYRK2 by TCGA project and GEO database.Research is carried out on gene expression levels, protein phosphorylation levels, gene mutation profiles and immune infiltration in various types of cancer. Through further study of DYRK2, it can be used to guide clinical practice and provide new ideas for cancer treatment.

## Results

### Gene expression analysis data

**Figure 1:**
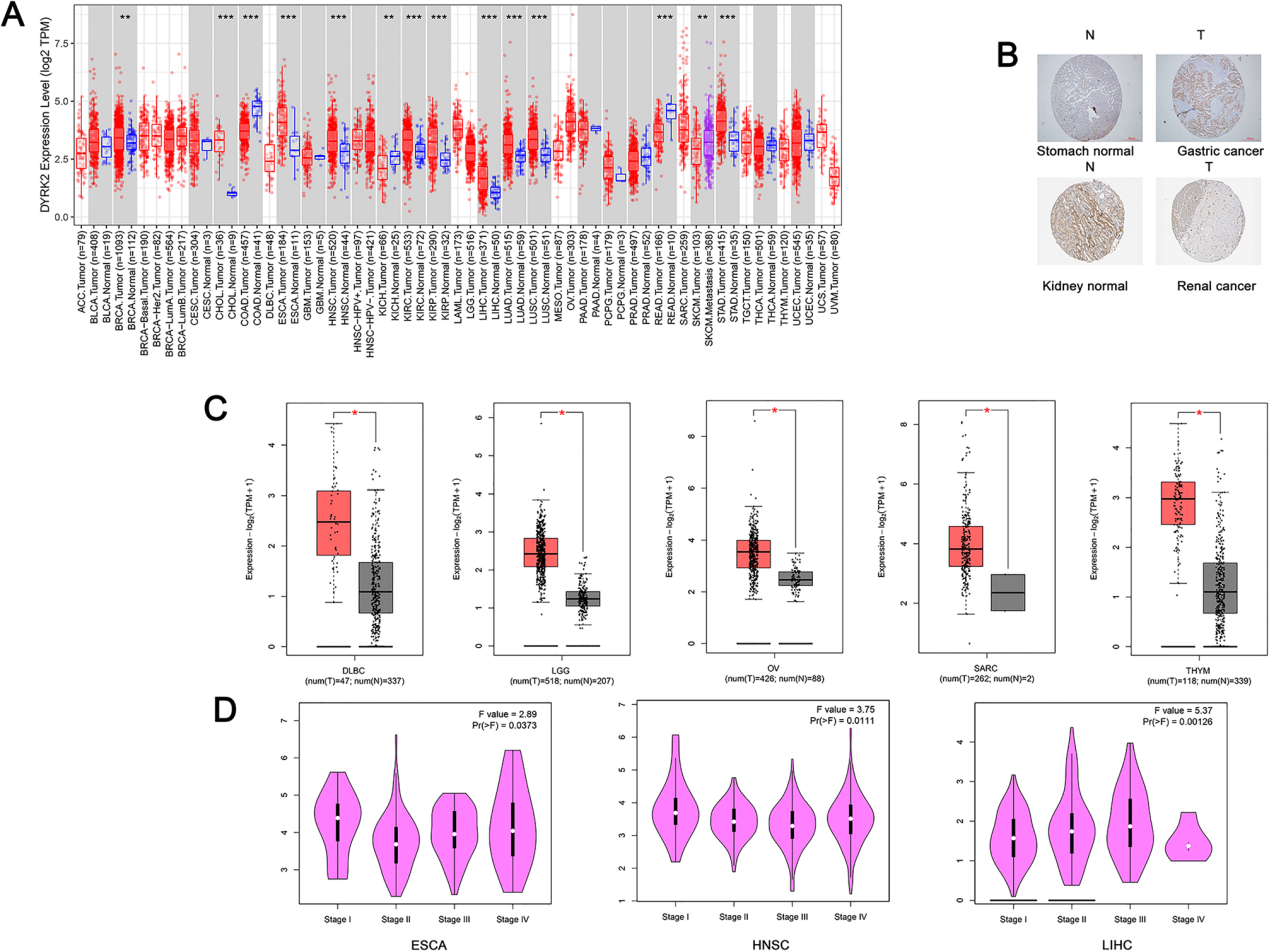
Expression levels of DYRK2 in various tumors and pathological stages. (**A**) Expression levels of DYRK2 in cancer and corresponding normal tissues in the database. *p < .05; **p < .01; ***p < .001. (**B**)Comparison of immunohistochemical images of DYRK2 in normal (left) and tumor (right) tissues of the stomach and kidney. (**C**) The differences in expression of DLBC, OV, LGG, SARC and THYM between cancer tissues and normal tissues in GTEx database were compared with box plots.* P < .05. (**D**) Expression levels of the DYRK2 were analysed by different pathological stages (stage I–IV) of ESCA, HNSC and LIHC. Log_2_ (TPM + 1) was used to represent the expression levels.

We first analysed the expression levels of DYRK2 in different blood cells and in normal human tissues. As shown in Fig. S1, based on a combined analysis of HPA (Human Protein Atlas), GTEx and FANTOM5 (Functional Annotation of the Mammalian Genome 5) data, the expression levels of DYRK2 were highest in liver and kidney, followed by tissues such as cerebellum, caudate and thyroid gland. DYRK2 was expressed in almost all tissues, and its RNA expression revealed overall low tissue specificity.RNA expression of DYRK2 in immune cells also showed low cell type specificity.Then,we used the TIMER2 method to analyze the expression levels of DYRK2 in tumor tissue and adjacent normal tissue in different cancers based on the TCGA database.As shown in Fig. 1A, the expression levels of DYRK2 in the tumor tissues of CHOL (Cholangiocarcinoma), ESCA (Esophageal carcinoma), HNSC (Head and Neck squamous cell carcinoma), KIRC (Kidney renal clear cell carcinoma), KIRP (Kidney renal papillary cell carcinoma), LIHC (Liver hepatocellular carcinoma), LUAD(Lung adenocarcinoma), LUSC (Lung squamous cell carcinoma), STAD (Stomach adenocarcinoma) (*P* < 0.001) and BRCA (Breast invasive carcinoma) (*P* < 0.01) were higher than the normal tissues of the control. However, the expression levels of DYRK2 in the tumor tissues of COAD (Colon adenocarcinoma), READ (Rectum adenocarcinoma) (*P* < 0.001),KICH (Kidney Chromophobe)(*P* < 0.01) were less than the normal tissues of the control.Immunohistochemical staining revealed that in gastric cancer, DYRK2 staining was deeper in the cancerous tissue than in the corresponding paracancerous tissue. This was in contrast to renal cancer(Fig. 1B).

For tumours without corresponding normal tissue in TCGA, we further assessed the diversities in DYRK2 expression between them and corresponding normal tissues by GTEx dataset.We further estimated the expression difference of DYRK2 between the normal tissues and tumor tissues of DLBC (Lymphoid Neoplasm Diffuse Large B-cell Lymphoma), LGG (Brain Lower Grade Glioma), OV(Ovarian serous cystadenocarcinoma),SARC (Sarcoma),and THYM (Thymoma) (Fig. 1C, P  < 0.05). However, we did not obtain significant differences in other tumors, such as ACC (adrenocortical carcinoma), TGCT (testicular germ cell tumor), or UCS (Uterine Carcinosarcoma) (*P* > 0.05) (Fig. S2).

The correlation between the expression of DYRK2 and the pathological stages of cancers, including ESCA(Esophageal carcinoma),HNSC(Head and Neck squamous cell carcinoma) and LIHC(Liver hepatocellular carcinoma) were analyzed by the GEPIA2 tool (Fig. 1D, all *P* < 0.05). However, in other cancers, we found no clear relevance (Fig. S3).

### Survival analysis data

**Figure 2:**
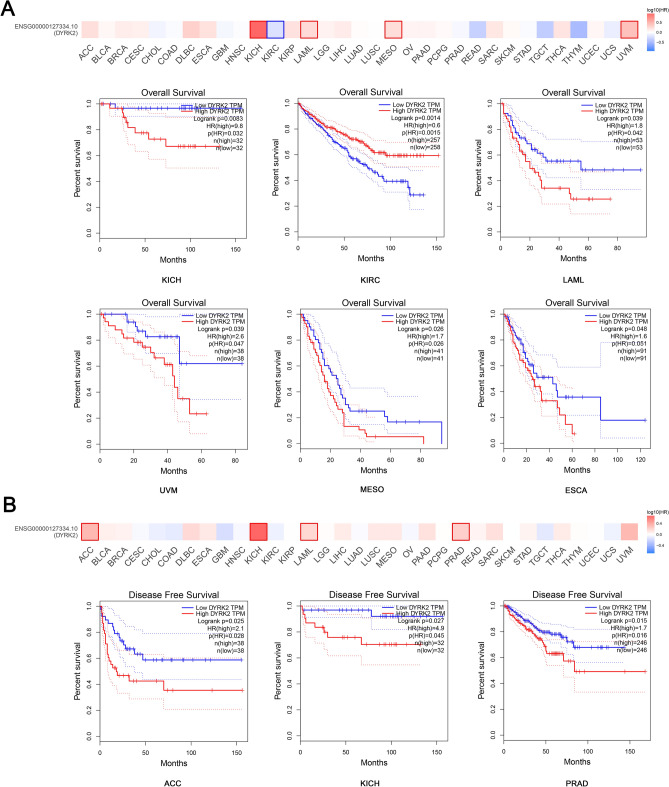
The relationship between the expression level of DYRK2 and the prognosis of tumor patients. The GEPIA2 website was used to analyze the overall survival (**A**) and disease-free survival (**B**) of different tumors according to the level of DYRK2 expression. Statistically significant survival plots were given.

The correlation between the expression level of DYRK2 and the prognosis of different cancer patients was obtained through TCGA and GEO datasets. As shown in Fig. 2A, in TCGA, high expression of DYRK2 predicted poorer overall survival in KICH (Kidney Chromophobe) (*P* = 0.0083), LAML (Acute Myeloid Leukemia) (*P* = 0.039), UVM (Uveal Melanoma) (*P* = 0.039), MESO (Mesothelioma) (*P* = 0.026) and ESCA(Esophageal carcinoma) (*P* = 0.048). Meanwhile, disease-free survival (DFS) data showed that high expression of DYRK2 suggested poor prognosis in TCGA cases in ACC (Adrenocortical carcinoma) (*P* = 0.025), KICH(Kidney Chromophobe) (*P* = 0.027) and PRAD (Prostate adenocarcinoma) (*P* = 0.015) (Fig. 2B). In contrast, low expression of DYRK2 was associated with poorer overall survival in KIRC(Kidney renal clear cell carcinoma) (Fig. 2A, *P* = 0.0014).

### Genetic alteration analysis data

**Figure 3:**
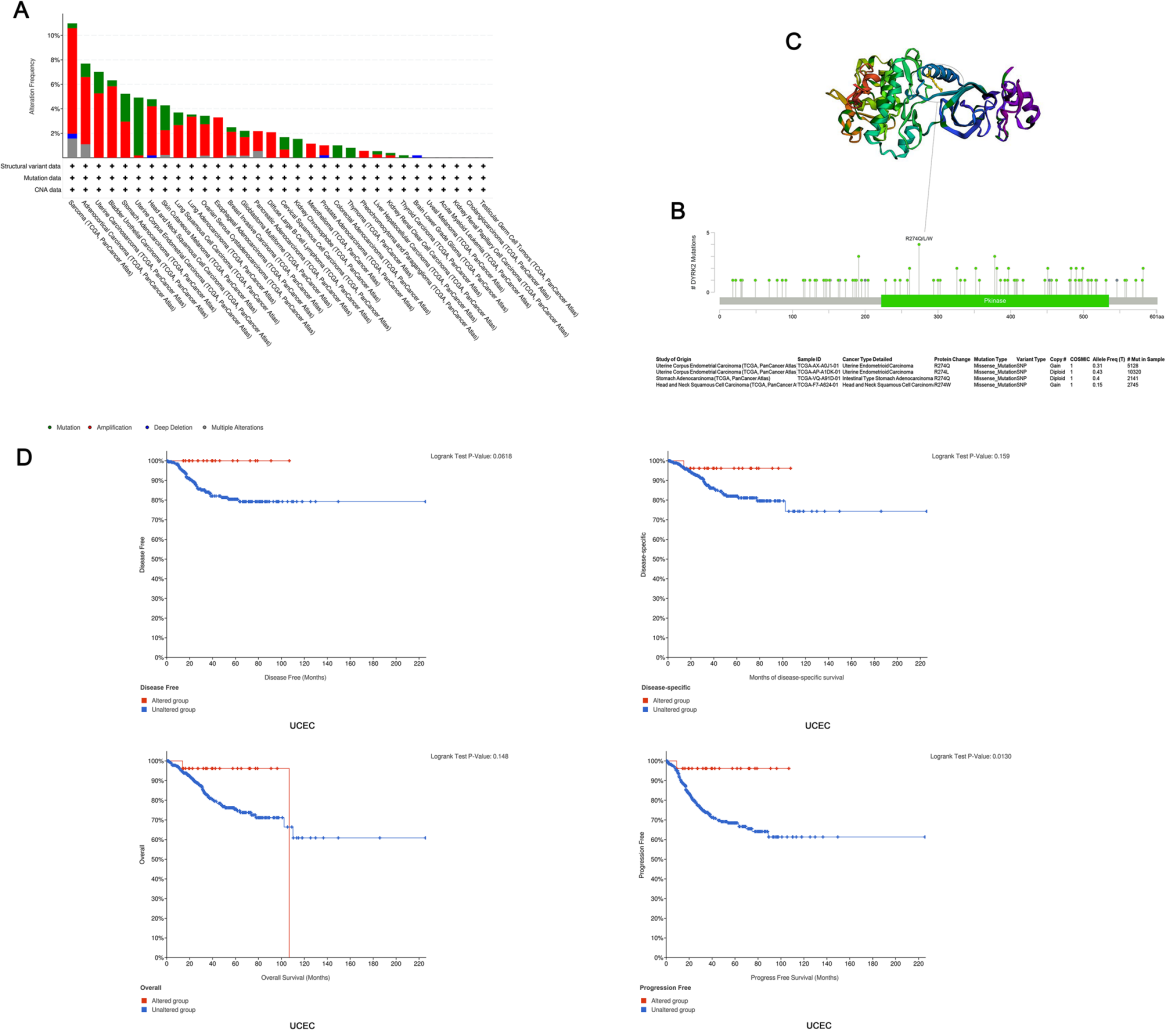
Mutation status of DYRK2 in TCGA tumors. The mutational status of DYRK2 in TCGA tumors were analysed by cBioPortal tool. The frequency of changes in mutation type (**A**) and mutation site (**B**) were shown. (**C**) Mutation sites in the structural domain of Pkinase (R274Q/L/W) were shown in 3D structure. (**D**) Correlations between UCEC mutational status and *OS* (overall survival), *DSS* (disease-specific survival), *DFS* (disease-free survival) and *PFS* (progression-free survival) were analysed by cBioPortal.

The progression of human cancer is accompanied by mutations in genes and for this reason we will investigate the relationship between mutations in the DYRK2 gene and tumors.We obtained a map of genetic alterations in DYRK2 in different tumour samples from the TCGA cohort. As shown in Fig. 3A, the highest frequency of alterations in DYRK2 (~ 5% frequency) was seen in patients with Uterine Corpus Endometrial Carcinoma, with 'mutations' being the predominant type. The “amplification” type was the dominant type in the sarcoma(> 8%) (Fig. 3A). The types, sites and case number of the DYRK2 genetic alteration exhibited in Fig. 3B. We identified missense mutations in DYRK2 as the predominant type of genetic alteration, while R274Q/L/W alterations in the Pkinase domain were ascertained in two cases of UCEC, one case of STAD and one case of HNSC (Fig. 3B). We could observe the R274 site in the 3D structure of the DYRK2 protein (Fig. 3C). In addition, we investigated the potential link between genetic alterations in DYRK2 and clinical survival prognosis in different cancer types.For example, the data of Fig. 3D indicate that UCEC cases with altered DYRK2 showed poor prognosis in progression-free (*P* = 0.013),but not disease free(*P* = 0.0618),overall (*P* = 0.148) and disease-specific (*P* = 0.159) survival, compared with cases without DYRK2 alteration.In hepatocellular carcinoma, altered DYRK2 showed poorer prognosis in progression-free (*P* = 3.795e−3) and disease free(*P* = 1.626e−3) survival. However, no correlation was found between DYRK2 alterations and clinical prognosis in breast cancer (Fig. S4).

### DNA methylation analysis data

**Figure 4:**
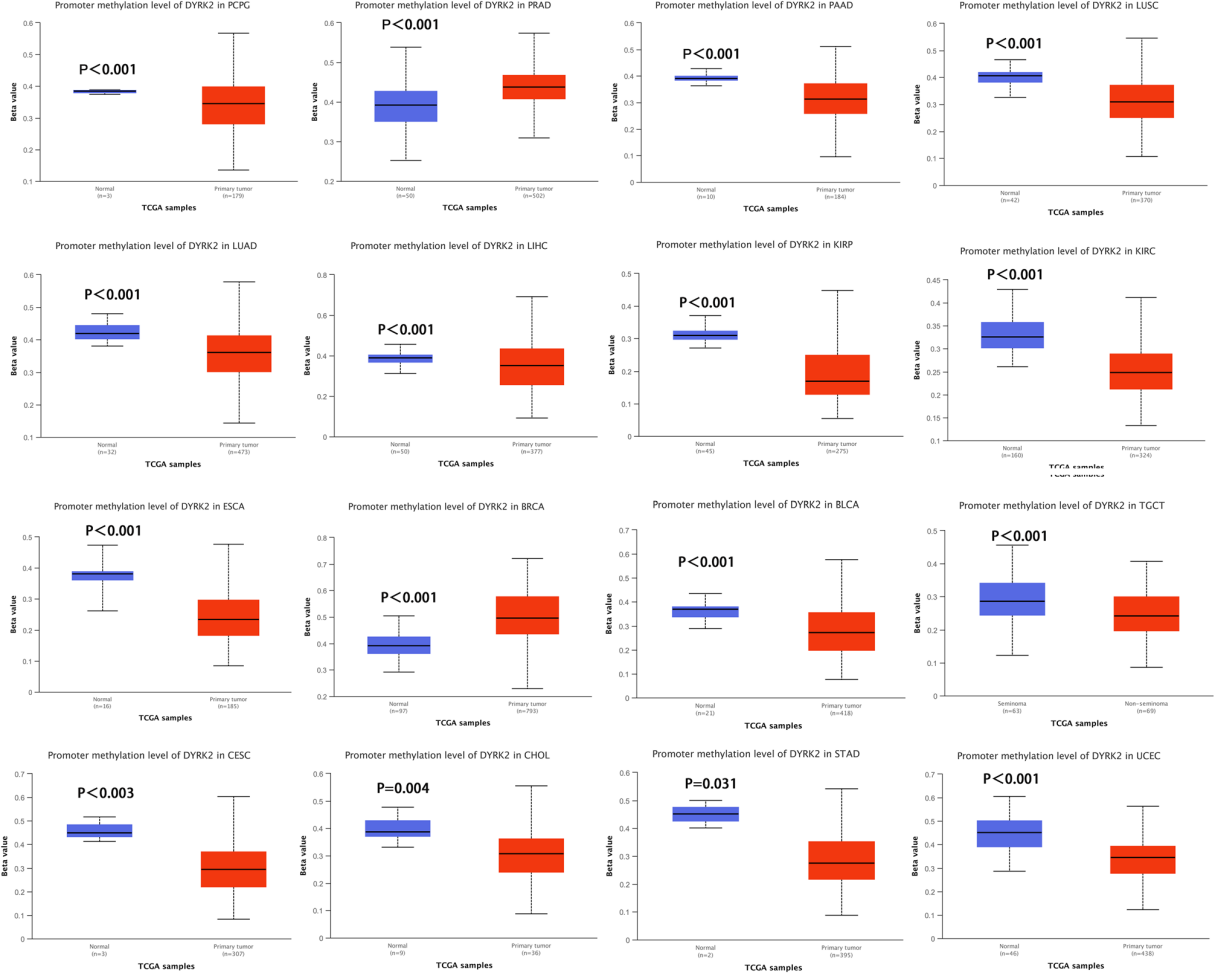
DNA methylation level of DYRK2 in different tumors. Promoter methylation levels of DYRK2 in different cancer types compared to normal adjacent tissues. The Beta value indicates level of DNA methylation ranging from 0 (unmethylated) to 1 (fully methylated).

Aberrant methylation can lead to tumorigenesis, so studying differences in methylation patterns can be used to distinguish tumors from benign tissues^19–21^. Methylation may cause or inhibit tumor formation. We used UALCAN to analyze the difference of DYRK2 promoter methylation level between tumor and adjacent normal tissues. Levels of DYRK2 promoter methylation in PCPG, PAAD, LUSC, LUAD, LIHC, KIRP, KIRC, ESCA, BLCA, TGCT, CESC, CHOL, STAD and UCEC were lower compared to corresponding normal tissues. On the other hand, levels of DYRK2 promoter methylation in PRAD and BRCA were higher than corresponding normal tissues (Fig. 4). Next, the potential association between DYRK2 DNA methylation and the pathogenesis of different tumors such as ESCA, STAD and LUAD was investigated by the MEXPRESS method. In Fig. S5, DNA methylation of DYRK2 was significantly correlated with gene expression on many probes.

### Protein phosphorylation analysis data

**Figure 5:**
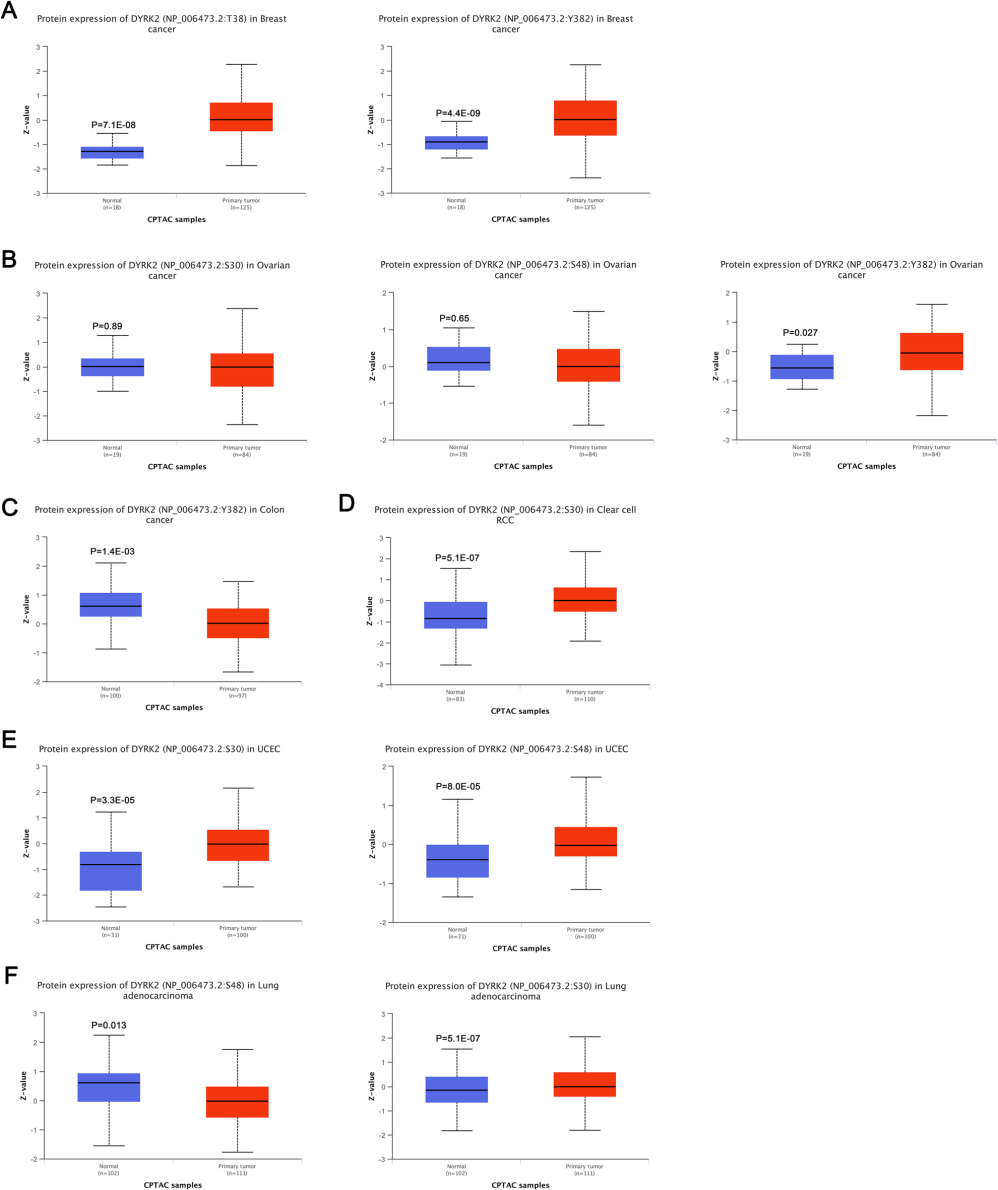
DYRK2 protein phosphorylation levels in the diversity of tumors. Comparison of the level of DYRK2 phosphoproteins (Y382, S30, and S48 sites) between normal tissues and primary tumor tissues (BRCA, OV, COAD, KIRC, UCEC and LUAD) (**A–F**).

We compared differences in DYRK2 phosphorylation levels between several cancers(BRCA, OV, COAD, KIRC, UCEC and LUAD) and corresponding normal tissues using the CPTAC dataset in Fig. 5. The Y382 locus exhibits a higher phosphorylation level in breast and ovarian cancer compared with normal tissues (Fig. 5A,B, all *P* < 0.05). Correspondingly, the S30 locus in Clear cell renal cell carcinoma, Uterine corpus endometrial carcinoma and Lung adenocarcinoma performed higher levels of phosphorylation compared to normal tissue(Fig. 5D–F, all *P* < 0.05).At the same time, it was found that increased phosphorylation levels at the S48 site of UCEC (Fig. 5E, all *P* < 0.05).

### Immune infiltration analysis data

**Figure 6:**
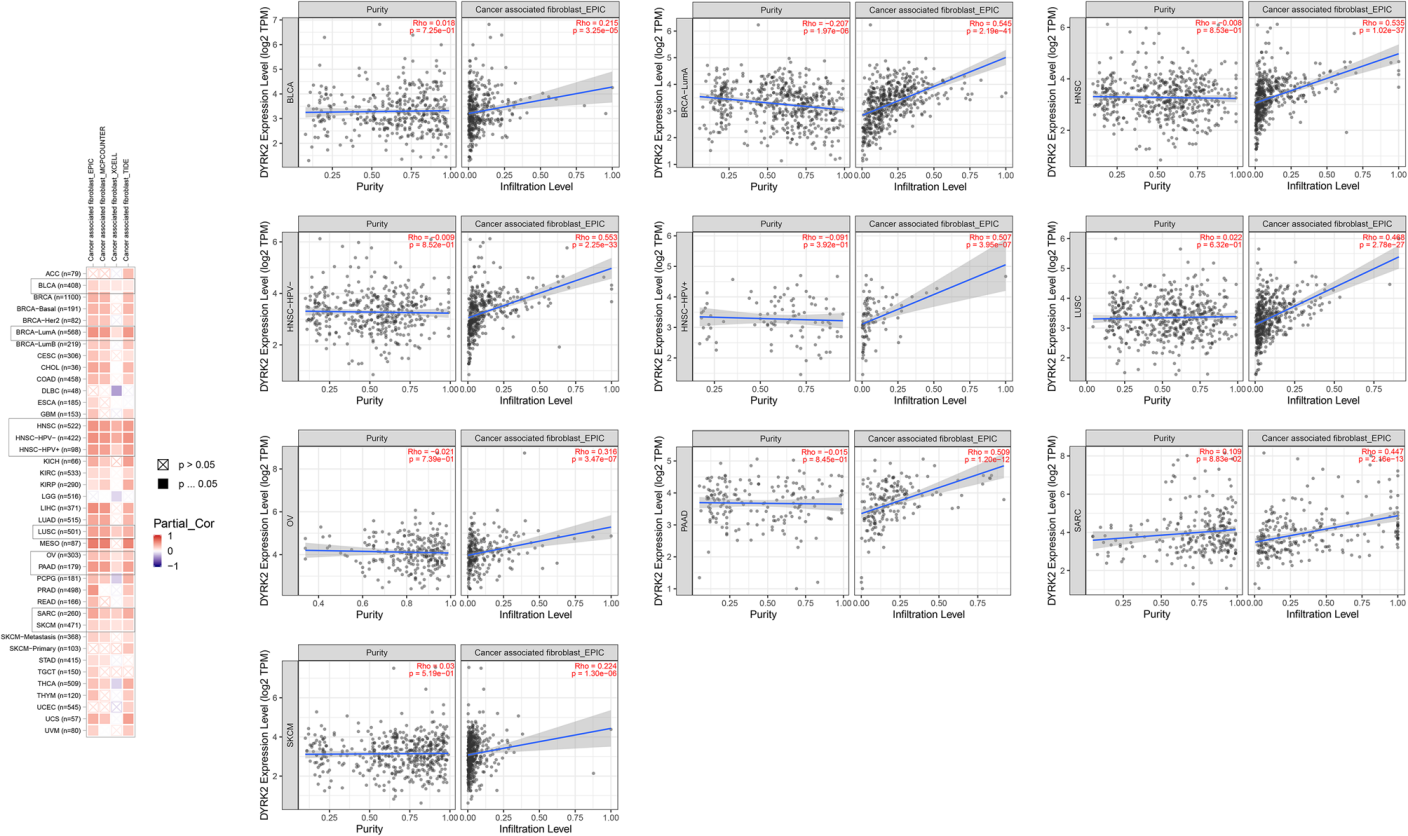
The relationship between the expression of DYRK2 and immune infiltration of cancer associated fibroblasts. A large number of algorithms were used to analyse the correlation between the expression levels of the DYRK2 and cancer-associated fibroblast infiltration levels. Positive correlations are indicated in red colour (0–1), while blue colour represents negative correlations (−1 to 0). P-value < 0.05 are considered to be statistically significant.

Tumor-infiltrating immune cells are an important part of the tumor microenvironment and participate in tumor initiation, progression or tumor cell metastasis ^22,23^. Cancer-associated fibroblasts in the tumor microenvironment stroma are involved in regulating the functions of various tumor-infiltrating immune cells ^24,25^. Therefore, it is of great significance to investigate the latent relationship between different immune cell infiltration levels and DYRK2 expression in TCGA of different cancer types through a series of algorithms. We found a statistically positive correlation between DYRK2 expression and tumor-associated fibroblast infiltration values in BLCA, BRCA-LumA, HNSC [HPV (Human papillomavirus) + / −],LUSC,OV,PAAD,SARC and SKCM (Fig. 6). Scatter plots of these cancers produced by an algorithm were drawn in Fig. 6. For example,we used EPIC algorithm to investigate the DYRK2 expression level in SARC,which is positively correlated with the infiltration level of cancer-associated fibroblasts (Fig. 6, RHO = 0.447, *P* = 2.16e−13) .

### Enrichment analysis of DYRK2-related genes

**Figure 7:**
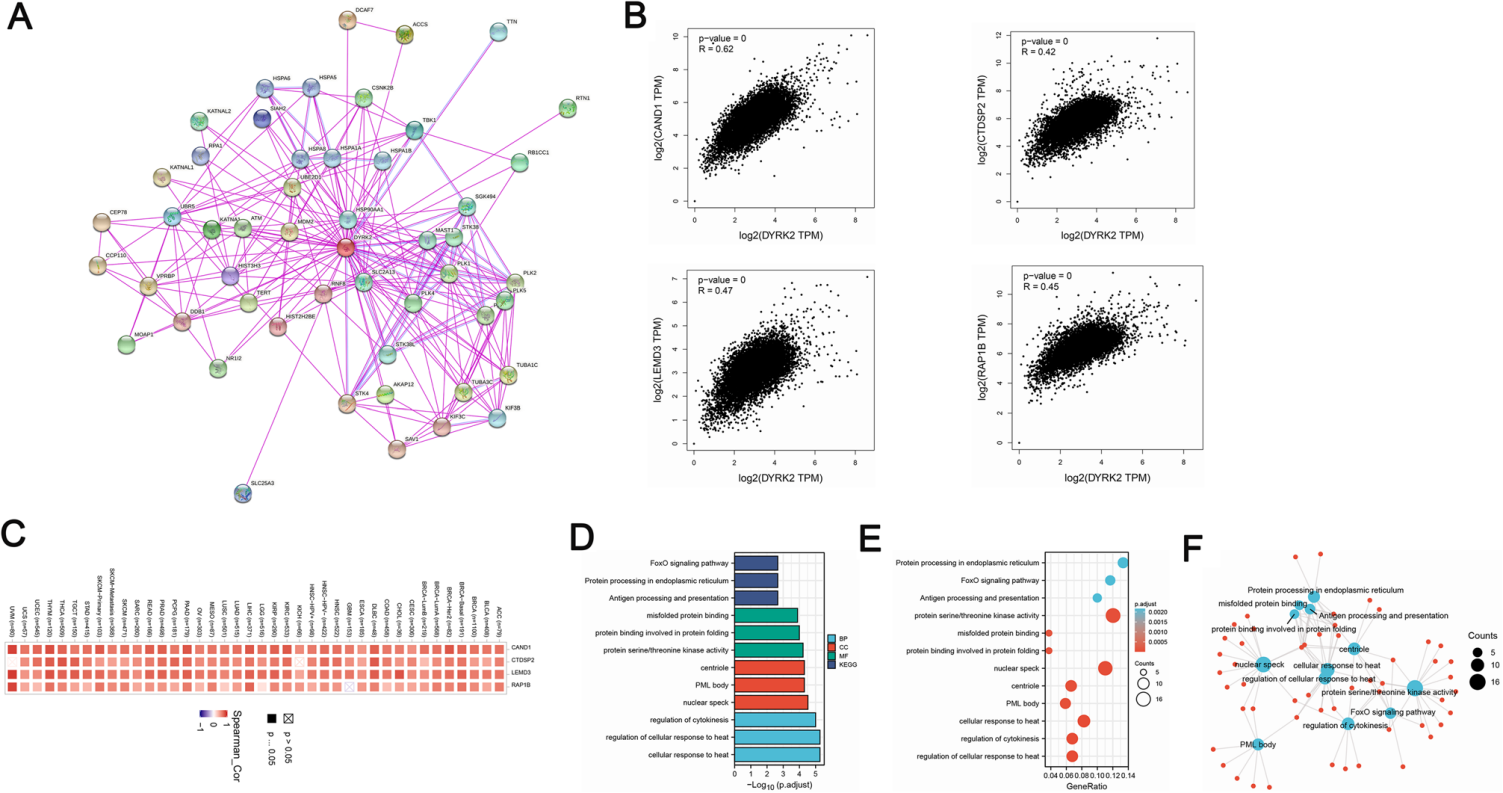
Enrichment analysis of DYRK2-associated genes. (**A**) The DYRK2-binding protein network was constructed by the STRING tool. (**B**) The top 100 DYRK2-related genes were obtained from the TCGA project using the GEPIA2 method, four target genes (CAND1, LEMD3, RAP1B, CTDSP2) were chosen and their expression correlations with DYRK2 were analysed. (**C**) The relationship between DYRK2 and these four genes in various types of cancer was shown by heat map. (**D–F**) GO/KEGG pathway analysis based on DYRK2 binding and interacting genes.

The molecular mechanisms of the DYRK2 gene in tumourigenesis are complex and we carried out a series of pathway enrichment analysis of DYRK2-binding proteins and expression-related genes. We obtained 50 experimental data-validated DYRK2-binding proteins using the STRING tool. A network diagram showed the interrelationships between the 50 proteins (Fig. 7A). The top 100 genes associated with DYRK2 expression were then selected in the TCGA database by the GEPIA2 tool. As shown in Fig. 7B, the DYRK2 expression level was positively correlated with CAND1 (R = 0.62), LEMD3 (R = 0.47), RAP1B (R = 0.45), CTDSP2 (R = 0.42) (all *P* < 0.001). A heat map showing the positive correlation between DYRK2 and the expression of these four genes in human cancers except in UVM, GBM, KICH (Fig. 7C). Combining these two data sets, we performed KEGG and GO enrichment analysis. The KEGG and GO data of Fig. 7D–F suggest that DYRK2 binding protein and DYRK2 expression-related genes were found to be associated with the FoxO signalling pathway through signaling pathway enrichment. This pathway regulates gene expression in cellular physiological events, including apoptosis, cell cycle control, glucose metabolism, resistance to oxidative stress and longevity. It is also involved in the development of cancer. In terms of molecular function, they have serine/threonine protein kinase activity can regulate different molecular pathways in normal and tumour cells and are involved in the development of cancer.At the cellular component level, these target genes are significantly associated with the PML body. PML is the central part of super-assembled structures called PML nuclear bodies (NBs)^26^. In human cancers, PML has been shown to play a dual role. The down-regulation of PML protein expression in the tissues of many cancer patients suggests that PML as a tumour suppressor initiates carcinogenesis when down-regulated. More and more studies are now showing that PML can act as a tumour initiator leading to carcinogenesis in chronic granulocytic leukaemia as well as in solid tumours such as glioma and breast cancer.The above indicates that PML may be used as a therapeutic target for the treatment of leukemia and solid tumors in the future.At the level of biological processes, DYRK2 and its related genes are involved in the cellular response to heat,which is essential for protection and recovery from cell damage. At the present day,proteostasis networks have emerged as promising targets for anti-tumour therapy. However,these genes are involved in the regulation of cytokinesis, thereby promoting tumour heterogeneity and cancer development. Recent studies have identified that induction of cytoplasmic division failure may become a promising antitumor therapy for a broad range of cancers, especially those characterised by rapid cell proliferation and polyploidy.

## Discussion

It has been reported that DYRK2 plays an important role in cell proliferation and apoptosis, epithelial mesenchymal transition (EMT) and cell cycle regulation^13–17^. However, several studies have reported a major oncogenic effect of DYRK2, and a few studies have determined that DYRK2 drives the development of cancer^27–29^. DYRK2 maintains protein stability in cancer cells by promoting proper protein folding or degradation. The transcription factor heat shock factor 1 (HSF1) is a master regulator of the proteotoxic stress response, supporting tumorigenesis by helping cancer cells cope with proteotoxic stress. In TNBC (triple negative breast cancer) cells, DYRK2 actively regulates the nuclear stability and activity of HSF1 by phosphorylation at ser320 and ser326^18^. Therefore, DYRK2 may play different functions to influence patient prognosis and could be a novel target for cancer therapy in different cancer types. However, we still little know about the involvement of DYRK2 in certain tumour types, or the role it plays in leading to tumourigenesis. So, our comprehensive approach includes a pan-cancer analysis of DYRK2 based on data from TCGA. We also collected, collated and analysed data using the CPTAC and GEO databases. Thus, the molecular features of DYRK2, such as its level of expression, tumor prognosis, phosphorylation and methylation levels, immunology and related signaling pathways, were investigated.

Through this study, we found that the expression level of DYRK2 in the tumor tissues of CHOL, ESCA, HNSC, KIRC, KIRP, LIHC, LUAD, LUSC, STAD and BRCA is higher than the corresponding normal tissues. However, the expression level of DYRK2 in the tumor tissues of COAD, READ, KICH is less than the corresponding normal tissues with lower expression. This demonstrated that DYRK2 may have distinct effects on different cancer types.

Through survival analysis, we concluded that high expression of DYRK2 was associated with poor overall prognosis in KICH, LAML, UVM, MESO and ESCA. Meanwhile, data from disease-free survival (DFS) analysis showed that high expression of DYRK2 was associated with poor prognosis in TCGA cases in ACC, KICH and PRAD. The results state clearly that DYRK2 is a potential biomarker to predict the prognosis of tumor patients.For example,DYRK2 has been less studied in ESCA, and high DYRK2 expression may predict poor prognosis through database analysis.

Gene mutation is an important cause of cancer occurrence and development. TCGA data showed that the DYRK2 gene was altered at different loci in distinct cancers, and in most cancer types, the alteration of DYRK2 gene was mainly amplified. What's more, this genetic alteration affects the prognosis of cancer patients. Abnormal DNA methylation is associated with tumorigenesis^19,30^. At the same time, we found that DYRK2 methylation plays a dual role, both carcinogenic and suppressive in some cancers.

It is not difficult to find a statistically positive correlation between DYRK2 expression and the estimated infiltration value of cancer-associated fibroblasts for cases of BLCA, BRCA-LumA, HNSC [HPV (Human papillomavirus) + / −], LUSC, OV, PAAD,SARC and SKCM. This better illustrates that DYRK2 is involved in tumorigenesis and development and plays an important role in the tumor microenvironment.

In summary, the first pan-cancer analysis of DYRK2 showed a statistical correlation between DYRK2 expression and clinical prognosis, DNA methylation, protein phosphorylation of clinical tumor samples, immune cell infiltration, and tumour mutation burden. This helps to understand the role of DYRK2 in tumorigenesis from the perspective of clinical tumor samples, and points out a new direction for cancer clinical treatment.

## Materials and methods

### Gene expression analysis

We used TIMER2 (tumor immune estimation resource, version 2, http:// timer.cistrome.org/) to seek the expression of DYRK2 in tumour tissues and adjacent normal tissues. However, some cancers showed no normal tissue,[e.g., TCGA-DLBC, TCGA-LGG], we applied the GEPIA2 (Gene Expression Profiling Interactive Analysis, version 2) tool (http://gepia2.cancer-pku.cn/#analysis) to obtain box plots of the GTEx (Genotype-Tissue Expression) database, setting P-value cutoff = 0.05, log_2_FC (fold change) cutoff = 1, and “Match TCGA normal and GTEx data”.Moreover, we got violin plots of the DYRK2 expression in different pathological stages (stage I-IV) of all TCGA tumors via GEPIA2 tool (http://gepia2.cancer-pku.cn/#analysis).

The CPTAC (Clinical Proteomics Tumor Analysis Consortium) dataset was analyzed for canceromic data and protein expression analysis by visiting the UALCAN website (http://ualcan.path.uab.edu/analysis-prot.html) to compare DYRK2 phosphorylated protein levels in primary tumors and normal tissues.

### Immunohistochemical (IHC) staining

To assess the differential expression of DYRK2 from the protein level, IHC images of DYRK2 protein expression in normal tissues and renal cell carcinoma were found and analyzed from HPA (Human Protein Atlas) (http://www.proteinatlas.org/). And the expression of DYRK2 in cancerous and paracancerous tissues was assessed by immuno-histochemical staining in gastric cancer.

### Survival prognosis analysis

We took advantage of the “Survival Map” module of GEPIA2, in order to acquire the OS (Overall survival) and DFS (Disease-free survival) survival data for DYRK2 in all TCGA tumors. High and low expression were divided by 50% cutoff value. The hypothesis test adopted the log-rank test and the survival plots were received from the “Survival Analysis” module of GEPIA2.

### Methylation analysis

We studied the difference in DYRK2 DNA promoter methylation levels between normal and tumor tissues through the "TCGA gene analysis" function of the UALCAN portal. The Beta value indicates level of DNA methylation.The DYRK2 DNA promotor methylation levels in some tumors were analyzed. MEXPRESS (https://mexpress.be/) serves as an online tool for visualizing TCGA gene expression, DNA methylation, clinical data, and their relationships^31^.In our study, we used this tool to find correlations between the expression of DYRK2 and promoter methylation levels in different cancer subtypes. Pearson correlation test selected the truncated value of *P* value < 0.05.

### Genetic alteration analysis

Through cBioPortal tool (https://www.cbioportal.org/), the data of protein structure change frequency, mutation type, mutation site information, CNA (copy number change) and 3D (three-dimensional) structure in all TCGA tumors observed in the "Cancer Types Summary" module were collected. Concurrently, we collected the data on the overall, disease-free, progression-free, and disease-specific survival differences in TCGA cancer cases with or without DYRK2 gene alteration.

### Immune infiltration analysis

After logging in to TIMER2 (http://timer.cistrome.org/), we went to the “immune” module, clicking on “gene”, entered “DYRK2”, and selected “cancer associated fibroblast” in “Immune Infiltrates”. The TIMER, CIBERSORT, CIBERSORT-ABS, QUANTISEQ, XCELL, MCPCOUNTER and EPIC algorithms were used to evaluate immune infiltration^32^.

### DYRK2-related gene enrichment analysis

In accessing the STRING website (https://cn.string-db.org/) for the protein–protein interaction network analysis, and the setting steps were as follows: meaning of network edges ("evidence"),active interactions sources (“experiments”),minimum required interaction score ["Low confidence (0.150)"], and max number of interactors to show (“no more than 50 interactors” in 1st shell) ^33^.

We obtained the top 100 genes associated with DYRK2 by GEPIA2 website. We then performed a paired gene–gene Pearson correlation analysis between DYRK2 and the selected genes. *P* values and correlation coefficients (R values) were revealed. KEGG and GO analyses were performed using the genes associated or interacting with DYRK2 from the previous analysis.

## Supplementary Information

Supplementary Figure S1.

Supplementary Figure S2.

Supplementary Figure S3.

Supplementary Figure S4.

Supplementary Figure S5.

Supplementary Legends.

## Data Availability

The experimental data will be available on the request.
