# Supplementary figures and images for: A pan-cancer analysis of the oncogenic role of dual-specificity tyrosine (Y)-phosphorylation- regulated kinase 2 (DYRK2) in human tumors
Source: Sci Rep. 2022 Sep 14;12:15419. doi: 10.1038/s41598-022-19087-7 (PMC9474874; doi:10.1038/s41598-022-19087-7)

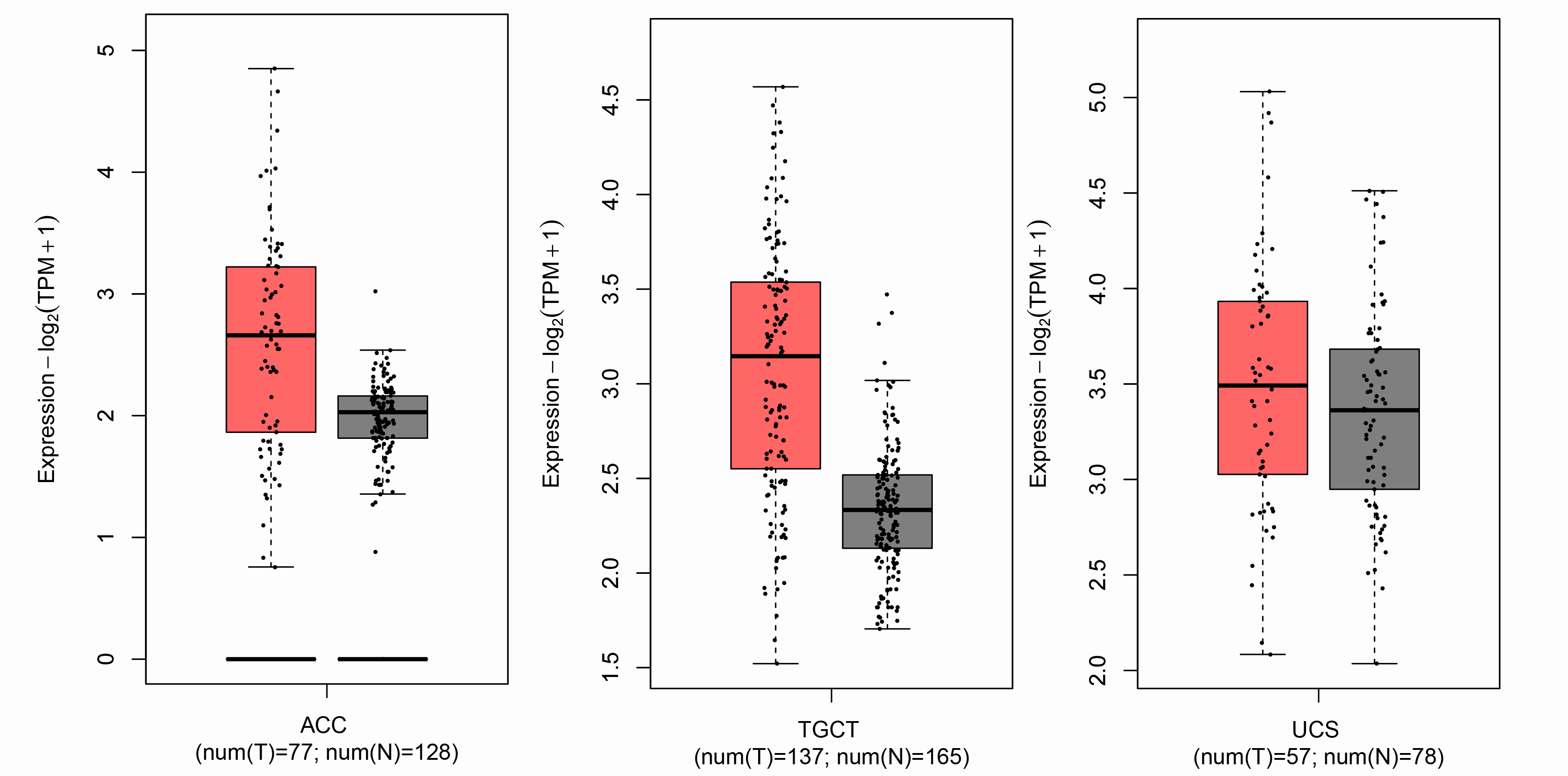

Supplement: Supplementary file 2 — Supplementary Figure S2. [file 41598_2022_19087_MOESM2_ESM.tif]

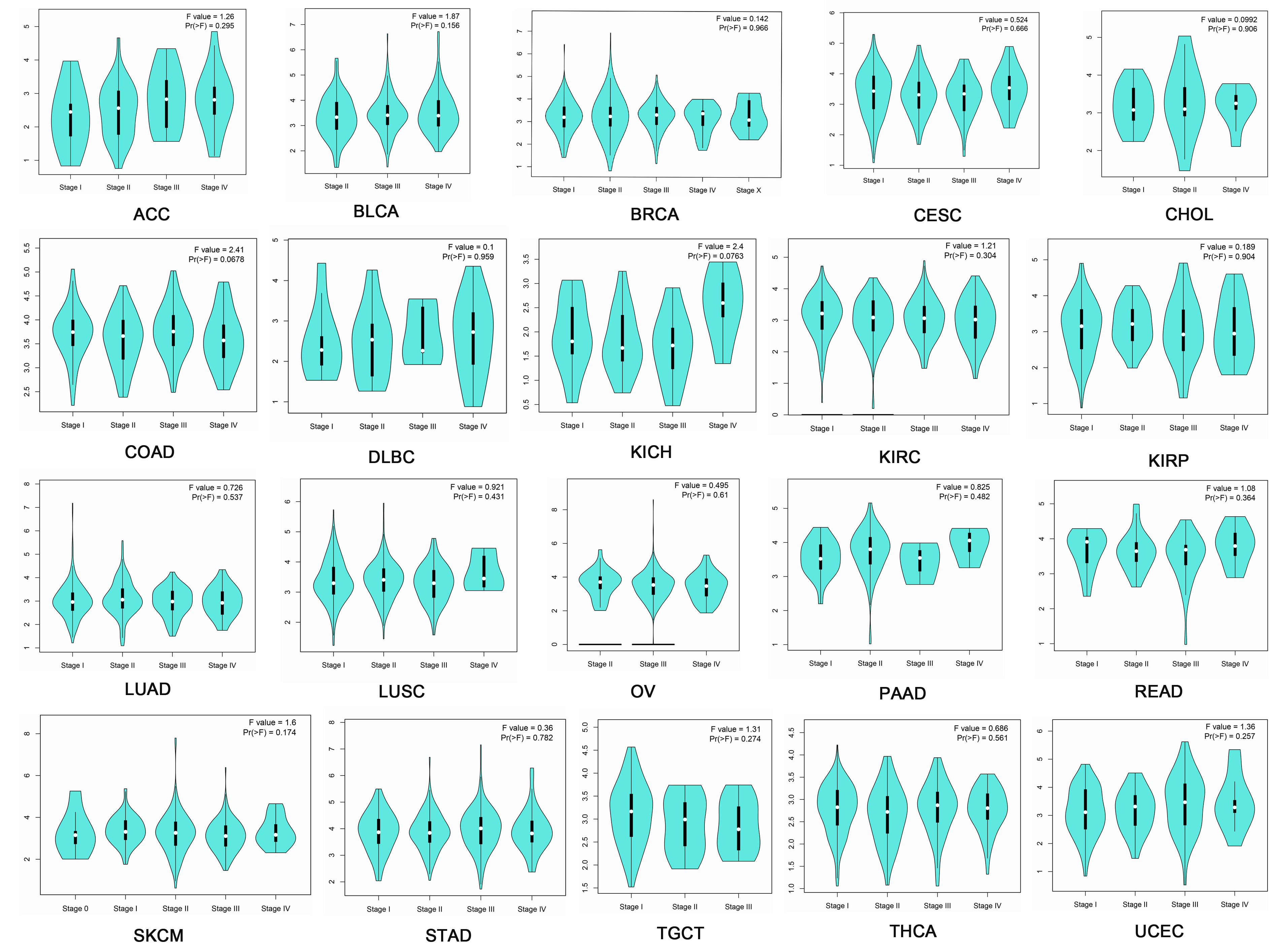

Supplement: Supplementary file 3 — Supplementary Figure S3. [file 41598_2022_19087_MOESM3_ESM.tif]
