# Supplementary material for: A pan-cancer analysis of the oncogenic role of dual-specificity tyrosine (Y)-phosphorylation- regulated kinase 2 (DYRK2) in human tumors
Source: Sci Rep. 2022 Sep 14;12:15419. doi: 10.1038/s41598-022-19087-7 (PMC9474874; doi:10.1038/s41598-022-19087-7)
Supplement: Supplementary file 6 — Supplementary Legends. [file 41598_2022_19087_MOESM6_ESM.docx]

**Supplementary material**

Supplementary Figure 1

The expression levels of DYRK2 in different tissues and cells under normal conditions. (A) The expression of DYRK2 in different tissues based on HPA, GTEx and FANTOM5 database; (B) The expression of DYRK2 in different immune cell types.

Supplementary Figure 2

Comparison of DYRK2 expression levels using GTEx database. The expression status of the DYRK2 in tumors(such as ACC, TGCT, UCS) was compared with normal tissues based on the GTEx database.

Supplementary Figure 3

Relationship between the expression level of DYRK2 and pathological stages of tumors. Relationship between the expression of DYRK2 and different pathological stages in different tumors (ACC, BLCA, BRCA , CESC, CHOL,COAD, DLBC, KICH, KIRC, KIRP, LUDA, LUSC,OV,PAAD, READ, SKCM, STAD, TGCT, THCA, UCEC).

Supplementary Figure 4

Applying Kaplan-Meier survival curve to studying the relationship between the expression of DYRK2 and prognosis. Kaplan-Meier survival curve was used to perform the survival analyses of breast cancer and liver cancer patients.

Supplementary Figure5

MEXPRESS visualisation of cases for ESCA,STAD and LUAD. These pictures showed how DYRK2 expression and promoter DNA methylation were significantly correlated.
